# A Pilot Study on the Reliability of Ultrasound-Based Assessment of Patella Diameter and Sulcus Angle

**DOI:** 10.3390/diagnostics12123164

**Published:** 2022-12-14

**Authors:** Isa-Maria Schlüter, Robert Prill, Aleksandra Królikowska, Caren Cruysen, Roland Becker

**Affiliations:** 1 Brandenburg Medical School Theodor Fontane, 14770 Brandenburg an der Havel, Germany; 2 Center of Orthopaedics and Traumatology, University Hospital Brandenburg/Havel, Brandenburg Medical School Theodor Fontane, 14770 Brandenburg an der Havel, Germany; 3 Faculty of Health Sciences Brandenburg, Brandenburg Medical School Theodor Fontane, 14770 Brandenburg an der Havel, Germany; 4 Ergonomics and Biomedical Monitoring Laboratory, Department of Physiotherapy, Faculty of Health Sciences, Wroclaw Medical University, 50-367 Wroclaw, Poland

**Keywords:** biomedical monitoring, knee, patella diameter, patellar dislocation, reliability, reproducibility of results, sulcus angle, ultrasonography

## Abstract

This pilot study aimed to determine the reliability of a newly developed ultrasound-based protocol for the assessment of patella diameter and sulcus angle. The diameter of the patella expressed in mm and the sulcus angle, expressed in degrees were measured in the right knee in 12 healthy participants (eight women and four men) in two separate sessions by two examiners (experienced rater and inexperienced rater) using ultrasonography according to a developed standardized protocol. The reliability was determined on the calculated intraclass correlation coefficient, ICC, expressed as a 95% confidence interval (lower bound, upper bound). For the patella diameter measurement, intra-rater and inter-rater reliability were good to excellent, with the ICC exceeding 0.836–0.998 and 0.859–0.997, respectively. The intra-rater and inter-rater reliability of the sulcus measurement was moderate to excellent, as the ICC amounted to 0.559–0.993 and 0.559–0.990, respectively. The reliability of both measures increased with the experience of the examiner. Therefore, it was determined that the newly developed protocol for an ultrasound-based assessment of patella diameter and sulcus angle is reliable. Further studies validating their clinical use should be carried out.

## 1. Introduction

Luxation of the patella is one of the most common knee joint conditions in the normal population, with an incidence of 5.8/100,000. Patella dislocations occur in 3% of all knee injuries [1]. In 10–17 years old teenagers, the incidence is even higher and exceeds 29/100,000 [2]. First-time patella dislocations show a probability of recurrence of up to 44% and a lifelong elevated risk of patellar instability [2]. Patella stability is given by active and passive stabilizers. Bony morphology, such as femoral torsion, the shape of the patella and trochlea, and the surrounding soft tissue, comprises the passive stabilizer [1]. From 30 to 100° of knee flexion, the patella is stabilized by the sulcus trochleae through which it is sliding [3]. The quadriceps femoris muscle is the most crucial active stabilizer. Close to extension, the passive and static factors have the essential stabilizing effect, while dynamic stabilizers are more critical above the 60° flexion [3]. Risk factors for patellar instability include trochlear dysplasia as well as tuberosity of the tibia to trochlear grove distance (TT-TG distance) or quadriceps angle (Q-angle), and family history, especially in relation to patellofemoral instability and trochlear shape [3,4]. Trochlear dysplasia is an important predisposing factor for idiopathic patella luxation [4,5] and one of the main aetiologic factors for early patellofemoral osteoarthritis [6]. The most frequently used classification of trochlear dysplasia is based on lateral radiographs, the Dejour classification [7]. Today, magnetic resonance imaging (MRI) or computed tomography (CT) are additionally used. The degree of severity is based on the magnitude of the sulcus angle as well as the trochlear shape. The sulcus angle is defined as the angle between the medial and lateral trochlear wall (the highest condyle point to the lowest point of the intercondylar groove). The physiological sulcus angle is supposed to be 137 ± 8° [4]. An angle greater than 145° defines trochlear dysplasia [7,8]. An increased sulcus angle increases the risk of patellar dislocation [9]. It is unclear to what extent the transverse diameter of the patella has an influence on mediolateral mobility and, thus, on its stability.

Reliable assessments with few risks for patients are needed for knee joint diagnosis [10]. As a method for determining the sulcus angle, sonography offers advantages regarding radiation exposure, costs and time savings. The method is content valid as the knee joint is directly assessed via imaging. However, a major disadvantage is that the results often depend on the examiner. The results can be influenced by the transducer’s handling and settings of the ultrasound device. Currently, no validated technique is available for measuring the patella diameter and sulcus angle using ultrasound. Therefore, the main aim of the present pilot study was to determine the intra-rater and inter-rater reliability of a developed ultrasound-based protocol for the assessment of patella diameter and sulcus angle. Additionally, the study indicated whether the reliability is influenced by examiner experience.

## 2. Materials and Methods

The pilot study was conducted in accordance with the Declaration of Helsinki’s ethics guidelines and principles. All the study participants were informed about the purpose and the approach to be used and signed their informed consent. The study used a repeated measure design. Prior to measurements, a test run was conducted to identify possible obstacles to the implementation and thus ensure a proper process, resulting in a low-biased test protocol for the trial.

### 2.1. Participants and Examiners

Twelve healthy volunteers (8 women and 4 men, mean age 24 ± 2) were included in the study. The characteristics are shown in Table 1. The inclusion criteria were healthy adults with no current musculoskeletal diseases. Therefore, participants with knee pain, limited mobility or swelling, recent knee injuries, or knee prostheses were excluded. Further exclusion criteria trochlear dysplasia or dysfunctional medial patellofemoral ligament.

Each of the participants participated in two separate measurement sessions. The interval between the sessions was 45 min. During both the first and the second sessions, the examination was performed independently by Examiner number 1 and Examiner number 2. Examiner 1 was an experienced examiner and a tutor for sonographic skills university tutorials. Examiner 2 was an inexperienced examiner with basic sonographic skills gained in university tutorials. Before starting the measurements, a training session was held to practice the measurement technique and correct handling of the ultrasound probe. For maximum standardization, both investigators underwent training by the study director regarding the measurement technique.

### 2.2. Measuring Procedure

Ultrasound (US) was performed using an ultrasound scanner by GE (LOGIQ 200 PRO Series) with a 7 cm, 7,5 MHz linear transducer as well as a silicon start-up length to offset the convexity of the patella. The start-up length reduces false echoes and image distortions as the gel-like viscosity is similar to human tissue, referring to sound velocity and impedance.

The examined person was lying supine with a slightly elevated upper body and knees straight in 0° extension. After palpation of the patella of the examined limb, the transducer was placed centrally on the kneecap in a transversal direction. First, the patella was measured at its widest transverse diameter. The measuring points were placed at the medial and lateral ends of the hyperechogenic bone surface. The transducer was angulated so that the measuring line runs parallel to the upper margin of the picture.

**Figure 1:**
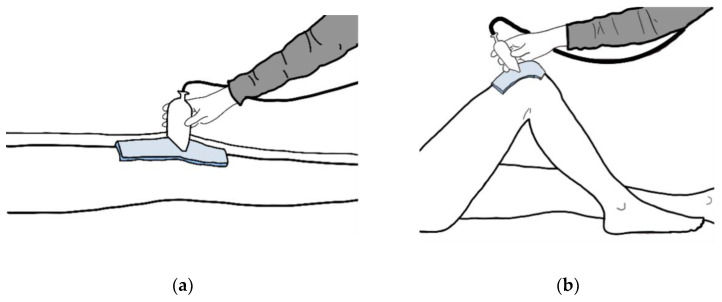
The ultrasound-based protocol for the assessment of (**a**) patella diameter; (**b**) sulcus angle.

For the measurement of the sulcus angle, the tested knee was positioned in 100° flexion. (Figure 1). The angle was measured in a suprapatellar transverse section. The transducer was held above the knee joint gap, and therefore was useful to mark it initially. It is then tilted until it represents the extension of the tibia. The measuring lines were placed on the hyperechogenic bone surface, not on the anechogenic cartilage.

**Figure 2:**
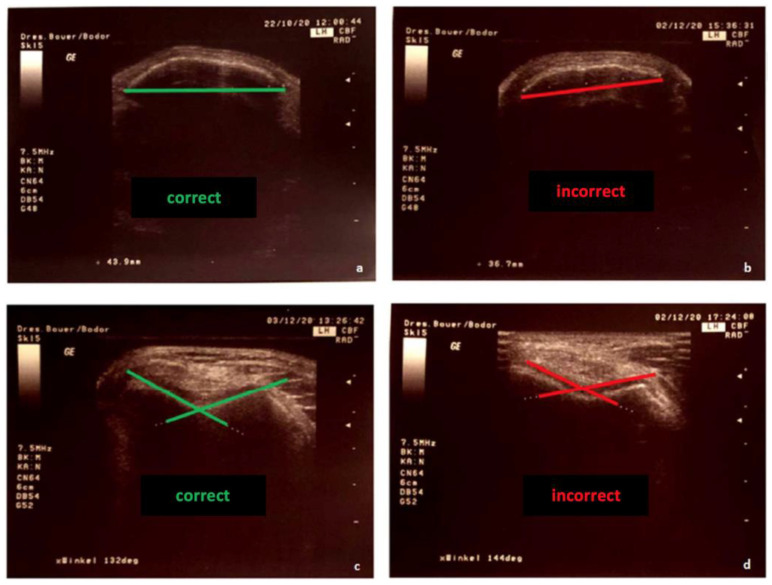
Image settings for sonographic measurement: (**a**) correct measurement of the patella diameter, (**b**) incorrect measurement plane, (**c**) correct positioning of the lines for the angle measurement, (**d**) incorrect positioning of the lines.

Therefore, the transducer was placed in a suprapatellar transverse plane using the extension of the femoral axis. The ultrasonic images were adjusted as necessary to obtain the maximum angle and echogenic cartilage, possible and a continuous strong echo line marking the bone (Figure 2). Correct and incorrect plane adjustments are also shown in Figure 2. A measuring protocol was developed for the standardization of the measurements. Examiner 1 started the measurement series by measuring the transverse patellar diameter, followed by Examiner 2. For blinding, values were hidden on the screen. The final adjusted image with the measurement result was printed. Then Examiner 1 positioned the tested knee in 100° flexion and measured the sulcus angle, followed by Examiner 2, also with covered values. The second measurement session took place after approximately 45 min, as analog to the first. Consistently in this study, the right knee joint was measured

### 2.3. Statistical Analysis

The statistical analysis was carried out using SPSS Statistics Version 28.0.1.0 (142) (IBM^®^ SPSS^®^ Statistics, Armonk, NY, USA) and Microsoft Office Excel 365 (Microsoft Corporation, Redmond, WA, USA). The studied group arithmetic mean (x) and standard deviation (SD) were calculated for patella diameter and sulcus angle. Outliers were included in the study. Subjects with missing data were excluded. According to performed Shapiro–Wilk test, the studied features were normally distributed. The reliability of performed measurements was based on the intraclass correlation coefficient (ICC) calculation according to the guidelines described by Shrout and Fleiss (1979) [11]. For assessing the reliability of the measurement carried out by the same examiner, so-called intra-rater reliability, two-way mixed-effects model, single measurement type and absolute agreement definition was used. When comparing the measurements between the two raters, we used a two-way random-effects model, measurement type and absolute agreement definition. The ICC interpretation was based on the estimated ICC’s 95% confidence interval (lower bound, upper bound). The ICC values smaller than 0.50 indicated poor reliability, values between 0.50 and 0.75 demonstrated moderate reliability, values between 0.75 and 0.90 indicated good reliability and values greater than 0.90 were considered to determine excellent reliability [12].

## 3. Results

### Intra-Rater Reliability

As presented in Table 2, the values of patella diameter and sulcus angle obtained during measurements performed by Examiner 1 were almost the same when comparing the first session to the second session. This is supported by excellent reliability, with the ICC ranging from 0.979 to 0.998 for the patella diameter and from 0.921 to 0.993 for the sulcus angle.

On the contrary, Table 3 presents the values of patella diameter and sulcus angle obtained during measurements performed by Examiner 2. The values obtained during the first session were comparable to those obtained in the second session; however, the reliability was at a lower level than in the case of an experienced examiner. The ICC ranging from 0.836 to 0.985 indicated good to excellent reliability for patella diameter measurements and the ICC ranging from 0.559 to 0.954 indicated moderate to excellent reliability for the measurements of sulcus angle.

Regarding patella diameter measurements, the reliability between Examiner 1 and Examiner 2 was good to excellent during the first measurement session and excellent during the second session. In the case of measurements of sulcus angle, the reliability between the two examiners was moderate to excellent during the first measurement session and from good to excellent during the second session. The interrater reliability was on a higher level during the second session when compared to the first (Table 4).

## 4. Discussion

### 4.1. Main Findings of the Study

The main aim of the present pilot study was to determine the intra-rater and inter-rater reliability of a developed ultrasound-based protocol for the assessment of patella diameter and sulcus angle. For the patella diameter measurement, intra-rater and inter-rater reliability were good to excellent. The intra-rater and inter-rater reliability of the sulcus measurement was moderate to excellent. However, it needs to be highlighted that the reliability of both measures increased with the experience of the examiner. It was noted that, when performed by an experienced examiner, both patella diameter and sulcus angle measurements are characterized by excellent reliability. What is more, during the second session, when the inexperienced examiner had already gained more experience, the between-examiners reliability of the measurements was also on a higher level than during the first session.

Our results are in accordance with a current meta-analysis showing that sonography is generally a reliable tool for the assessment of knee joint arthritis [13] and also for trochlear cartilage thickness [14]. Experience in ultrasound diagnostics also seems to be an essential issue concerning accuracy. Examiner 1 was an ultrasound tutor and therefore had more experience in sonography. In addition, he had drafted the measurement instructions so that the measurement technique may already have been better mastered. This is in accordance with findings in the field of traumatology, where a Focused Assessment Sonography for Trauma (FAST) for at least two days is strongly recommended [15].

Studies show that reliability can be further improved if the mean of two or three measurements is used to evaluate reliability [16,17]. This should be considered in further reliability studies on the examined methods of patella diameter and sulcus angle measurements. The form of blinding of the examiners was critical in this study. Blinding the examination would mean that the examiner only sees the knee joint but not the whole participant. In addition, it would be useful if the second measurement session carried out by Examiner 1 and Examiner 2, i.e., the so-called retest, took place on a different day. However, the measurement was carried out on the same day due to the general conditions. The measurement values were taped off on the screen to keep the results as objective as possible so that the examiners were not subjected to any influence. Only the study leader was able to see the parameters on the print and transferred them directly into the Excel table. In addition, a longer time interval would make sense in order to keep the memory effect of the examiners and, thus, the apparent reliability as low as possible. However, this always carries the risk of losing the presence of participants for the second measurement session.

### 4.2. Limitations of the Study

An important limitation for further generalization is the sample size of this study due to the nature of its pilot character. For reaching high method standards, as nowadays recommended for orthopedic trials [18,19], funding is usually needed and the value of the study must be proven in advance. Whenever possible, new studies should be justified through high standard Systematic Reviews showing the need for further studies on the topic [20,21,22]. This approach is called Evidence Based Research [23], leading to less redundant studies. If this reliable data is not available, Pilot testing is often needed to reduce waste. Initial pilot testing with healthy subjects, is common practice as justification and data for sample size calculation are needed in larger trials. This has also been carried out by the authors in various fields of orthopedic research areas and remains a feasible way to reduce waste and unpromising involvement of patients [24,25]. Still, generalizability of pilot testing results is partially questionable as sample size and setting are often likely to be biased.

### 4.3. Potential Future Research

Reliability is one aspect of diagnostic test accuracy and is relevant for new technologies in orthopedic research, such as sensor-based assessments or modern approaches imaging technology [26,27,28]. Still, the next step in the validation of the measurement method would be to test its accuracy by comparing the values determined using ultrasonography with those determined using a former validated procedure as a reference test. The gold standard for measuring the sulcus angle is the CT image. In addition, data collection with more experienced examiners in the field of musculoskeletal sonography would be interesting. Furthermore, other secondary outcomes could be evaluated such as the correlation of patellar diameter with patellar mobility or stability. For example, parallel testing of measurements using the new Patellometer device [29] on the mediolateral patella shift would be interesting to estimate how much sulcus angle is likely to explain medio-lateral shift. Providing more insights into the correlation of sulcus angle, patella movement, pain and function, we might be able to draw more conclusions in terms of topic like patella luxation or anterior knee pain and total knee arthroplasty. 

## 5. Conclusions

This study shows that ultrasound seems to be a reliable tool for the assessment of patella diameter and sulcus angle. A study with a bigger sample should be set up to evaluate ultrasound as a valid and reliable method for measuring the transverse patellar diameter and sulcus angle. If these reliability values are confirmed in future studies, this measurement method could be implemented in clinical diagnostics.

## Figures and Tables

**Table 1:** Demographic and clinical characteristics of the study population.

Characteristics	Distribution (*n* = 12)
Female *	8 (67%)
Male *	4 (33%)
Age (years)	24 ± 2
Body height (cm)	174.5 ± 8
Body weight (kg)	67 ± 11
BMI	19.13 ± 2.67
Participants with patella luxation *	1 (8,3%)

Reported as mean ± standard deviation, values marked with * shown (%).

**Table 2:** Results of the patella diameter and sulcus angle measured in the right knee by the experienced examiner during the first and second measurement sessions. The intra-rater reliability for the experienced examiner.

Intra-Rater Reliability; Examiner 1		
	Patella Diameter (mm)	Sulcus Angle (°)
Session 1	x = 40.50 ± 2.81	x = 131.50 ± 7.03
Session 2	x = 40.52 ± 2.81	x = 131.33 ± 7.52
ICC	0.994 (0.979; 0.998)	0.976 (0.921; 0.993)

Values expressed as arithmetic mean (x); standard deviation (±), intraclass correlation coefficient (ICC). The ICC was expressed as intraclass correlation and 95% confidence interval (lower bound, upper bound).

**Table 3:** Results of the patella diameter and sulcus angle measured in the right knee by the inexperienced examiner during the first and second measurement sessions. The intra-rater reliability for the inexperienced examiner.

Intra-Rater Reliability; Examiner 2		
	Patella Diameter (mm)	Sulcus Angle (°)
Session 1	x = 40.36 ± 2.69	x = 132.08 ± 7.27
Session 2	x = 40.54 ± 3.01	x = 132.58 ± 7.08
ICC	0.948 (0.836; 0.985)	0.849 (0.559; 0.954)

The values expressed as arithmetic mean (x); standard deviation (±), intraclass correlation coefficient (ICC). The ICC was expressed as intraclass correlation and 95% confidence interval (lower bound, upper bound).

**Table 4:** Reliability between patella diameter and sulcus angle measurements carried out by experienced and inexperienced examiners.

Reliability between Examiner 1 and Examiner 2		
	Patella Diameter (mm)	Sulcus Angle (°)
Session 1	0.956 (0.859; 0.987)	0.856 (0.578; 0.956)
Session 2	0.989 (0.964; 0.997)	0.962 (0.805; 0.990)

The values expressed as arithmetic mean (x); standard deviation (±), intraclass correlation coefficient (ICC). The ICC was expressed as intraclass correlation and 95% confidence interval (lower bound, upper bound).

## Data Availability

All raw data from this study are available upon request. Please contact isa-maria.schlueter@mhb-fontane.de for all inquiries.
